# Measuring In-Vivo Foot Perfusion Distal to a Near-Circumferential Negative Pressure Wound Therapy Dressing via Thermal Imaging

**DOI:** 10.7759/cureus.17720

**Published:** 2021-09-04

**Authors:** Dylan Singh, John P Livingstone, Jacob Lautze, Patrick C Murray

**Affiliations:** 1 Medicine, John A. Burns School of Medicine, University of Hawaii, Honolulu, USA; 2 Orthopedic Surgery, Queen's Medical Center, Honolulu, USA; 3 Medicine, Homer Stryker School of Medicine, Western Michigan University, Kalamazoo, USA; 4 Orthopedic Surgery, University of Hawaii, Honolulu, USA

**Keywords:** delayed wound healing, advanced wound care, wound healing, orthopedic surgery, closed incisional negative pressure wound therapy, incisional negative pressure wound therapy, wound vac, perfusion, blood flow, dressing

## Abstract

Background

Negative pressure wound therapy (NPWT) has been shown to promote the healing of acute and chronic wounds. In our previous study, we demonstrated that a near-circumferential NPWT dressing provided “lift-off” on an in-vitro extremity model resulting in decreased pressure. We hypothesized that this decrease in pressure may increase perfusion distal to the NPWT dressing by increasing lymphatic drainage and venous flow.

Methods

In this study, we tested if a near-circumferential NPWT dressing caused any appreciable skin movement around the dressing. We then used a thermal imaging camera to test if there was an increase in perfusion to the foot when a near-circumferential NPWT dressing was placed around the lower leg and tested at various negative pressures. Finally, we wanted to see if an artificial “lift-off” mechanism would lead to an increase in perfusion.

Results

The skin was noted to stretch between the short ends of the NPWT dressing, consistent with our previously described “lift-off” mechanism. However, there was no correlation between negative pressure and perfusion to the foot in the other experiments.

Conclusion

This study demonstrated that a near-circumferential NPWT dressing may not have any appreciable effects on perfusion when applied on a healthy patient, however, future studies are needed to determine if similar results would be seen on a traumatized or otherwise compromised extremity.

## Introduction

In 1997, Morykwas et al found that blood flow increased fourfold when applying 125mmHg of sub-atmospheric pressure to a wound in a pig model via negative pressure wound therapy (NPWT) [1]. These promising results pioneered the field of NPWT, and today, NPWT is often used to stabilize the wound environment, stimulate the growth of new tissue, and potentially assist in infection prevention and removal of bacterial debris [2-4].

Numerous studies have demonstrated an increase in perfusion directly under or around negative pressure wound therapy (NPWT) dressings. Timmers et al found that cutaneous blood flow, measured with laser doppler probes, increased perfusion at pressures as low as -300mmHg under the NPWT dressing [5]. Muenchow et al used laser spectrometry on the thighs of healthy patients and found that there was increased blood flow, capillary venous oxygen saturation, blood flow velocity, and hemoglobin levels directly under the NPWT dressing [6]. Other studies have measured blood flow around the wound edges and have shown increased microvascular blood flow at 2.5cm away from the wound edge, but decreased flow at 0.5cm from the wound edge, and no changes seen as far as 5cm away. It is theorized that the traction on surrounding tissues leads to a pulling force on the wound edge which dilates blood vessels and increases perfusion [7-11].

Although there is an abundance of literature demonstrating an increase in perfusion with NPWT, several studies have questioned their validity. Kairinos et al questioned the validity of laser doppler in predicting perfusion, rather than just measuring blood velocity. Since velocity increases as the vessels narrow, a higher velocity detected by laser doppler may not necessarily equate to increased tissue perfusion. Recent studies using other measurement methods including a laser doppler and Stryker SPY ELITEÒ thermal imaging system (Stryker, Kalamazoo, USA) have demonstrated either no change or a decrease in perfusion [12-17]. One study showed that the level of negative pressure can affect perfusion as well. Ichioka et al placed an NPWT dressing on an animal model and saw increased flow in microvasculature at -125mmHg but decreased flow at -500mmHg [10]. Overall, the literature is quite conflicting regarding the effect of NPWT on perfusion both deep to and around the NPWT dressing.

In this study, we followed up on our previous report where we demonstrated that a near-circumferential dressing which encircles about 2/3rds of the extremity led to decreased pressure in an extremity analogue and provided a “lift-off” force in vitro. This “lift-off” force is thought to occur as the NPWT dressing decreases in length and stretches the portion of the extremity that is not covered by the dressing. This leads to an increase in volume and a decrease in the pressure of the extremity. We hypothesized that these forces may be able to dilate vessels and increase perfusion in an extremity. We also hypothesized that this may improve venous return and lymphatic drainage thereby decreasing edema distal to the NPWT dressing [18]. Here, we aimed to determine if the “lift-off” mechanism we found in our previous study would have any clinical significance when applied in vivo. First, we tested if the NPWT dressing caused any appreciable skin movement around the dressing. Second, we used a thermal imaging camera to test if there was an increase in perfusion to the foot when a near-circumferential NPWT dressing was placed around the lower leg and tested at various negative pressures. Finally, we tested if an artificial “lift-off” mechanism could lead to an increase in perfusion.

## Materials and methods

This study consisted of two experiments that were performed on one of the authors of this study. This study did not require Institutional Review Board approval. Both experiments used a Prevena™ Plus Customizable Dressing by 3M + KCI (3M, Saint Paul, USA) which was applied per the manufacturer guidelines with the exception of not utilizing the sealing strips on the short edges of the dressing. These were omitted since, in our experience, they are not needed to maintain a proper seal and are very rarely used in our institution. The dressing was applied 2/3rds of the way around the extremity in each experiment since this was found to lead to the greatest decrease in pressure when tested on our previously published in-vitro study [18].

**Figure 1:**
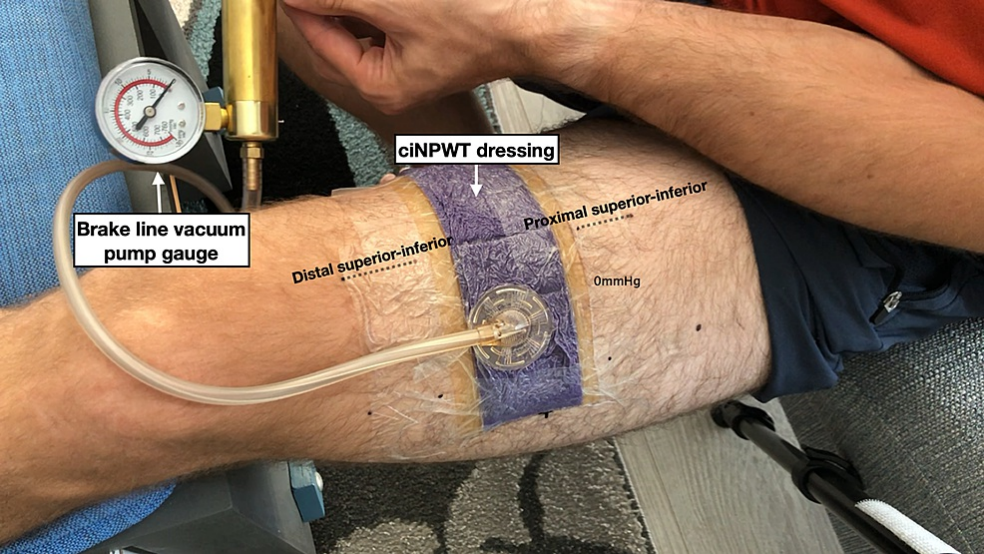
The NPWT dressing was applied to the thigh in a near-circumferential fashion. This image was taken anterior to the thigh so the distance between the markers on the anterior thigh could be measured. The brake line vacuum pump gauge was included in these images so the negative pressure could be monitored throughout this experiment. NPWT: negative pressure wound therapy

**Figure 2:**
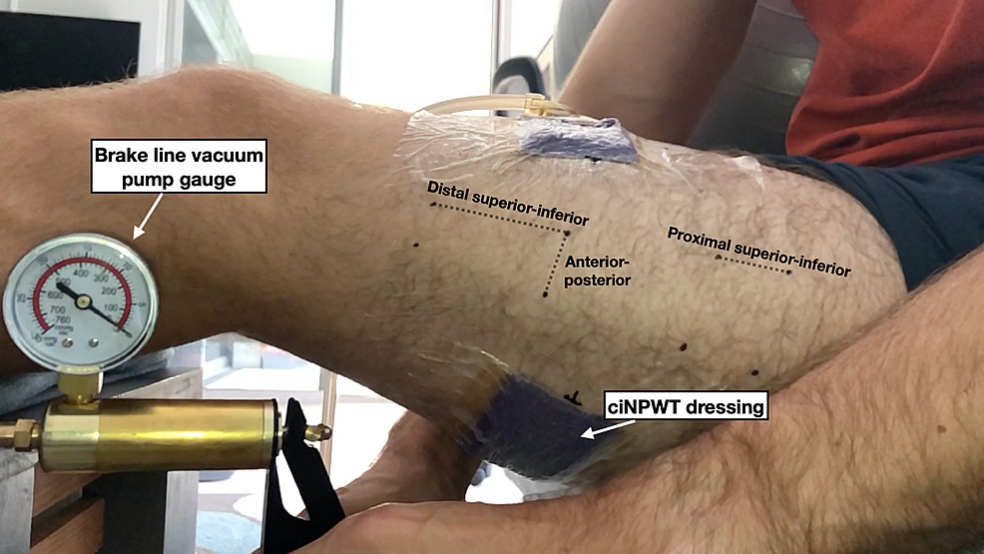
This image was taken lateral to the thigh so the distance between the markers on the lateral thigh could be measured.. ciNPWT: closed incisional negative pressure wound therapy

In the first experiment, the NPWT dressing was applied 2/3rds of the way around the thigh leaving the remaining third uncovered. Care was taken to ensure that the remaining third was not covered by any adhesive drapes. The thigh was chosen for this experiment because it offered the greatest surface area for a near-circumferential NPWT dressing. This was thought to provide the best chance of noting any changes in skin motion after the dressing was under negative pressure. Several dots were marked along the thigh with a permanent marker to serve as a grid by which we could measure skin motion (Figures 1-2). An iPhone X (Apple Inc, Cupertino, USA) was mounted to a tripod to record a video of the thigh as negative pressure was applied to the dressing. A handheld brake line vacuum pump was used to generate a wide range of negative pressures (0 to -380mmHg) that exceed the range of the manufacturer’s negative pressure system (-75 to -250mmHg). The negative pressure gauge on the brake line pump was included in the video so images could be captured from the video at varying negative pressures for further analysis. The distance between the dots on the thigh around the NPWT dressing was analyzed from these images with ImageJ (National Institutes of Health, Bethesda, USA) at negative pressures ranging from 0 to -380mmHg. The video was recorded lateral to the thigh as well as anterior to the thigh to measure the skin changes from different angles.

**Figure 3:**
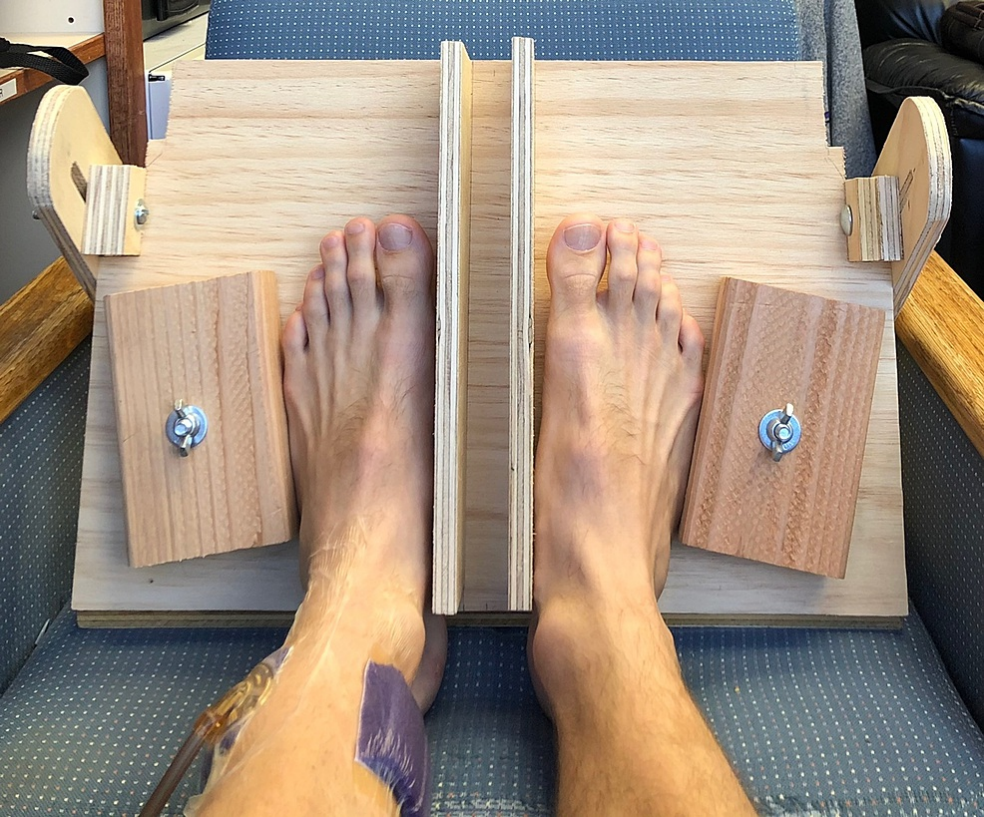
This custom jig held the feet in place to eliminate any motion artifact during the experiment. The NPWT dressing was applied to the left lower leg, proximal to the ankle. NPWT: negative pressure wound therapy

**Figure 4:**
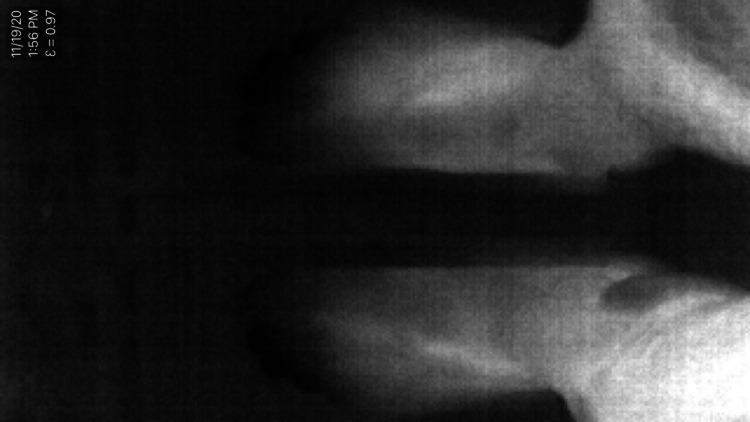
Thermal image was taken in greyscale using the Seek Thermal CompactPRO with white representing the highest temperatures and black representing the lowest temperatures. The NPWT dressing is on the left lower leg. NPWT: negative pressure wound therapy

In the second experiment, the NPWT dressing was applied around the distal lower leg just proximal to the ankle (Figure 3). The contralateral leg had no NPWT dressing as a control. The distal lower leg was chosen for this experiment because the temperature of the foot can vary significantly under different conditions and can be easily monitored with a thermal imaging camera. If our hypothesis that a near-circumferential NPWT dressing can improve perfusion distal to the dressing was true, we would expect to see an increase in the temperature of the foot as negative pressure was applied to the dressing. Both feet were secured in a custom platform so the feet would not move throughout the course of this experiment (Figure 3). A Seek Thermal CompactPRO (Seek Thermal Inc., Santa Barbara, USA) for iOS was used with an iPhone X mounted to a tripod to record thermal images of both feet throughout the experiment. The thermal images were taken in greyscale with white representing the highest temperatures and black representing the lowest temperatures (Figure 4). A thermal image was taken every two minutes as the dressing underwent 20-minute cycles of negative pressure being off, on, off, and on. This experiment was performed at -25mmHg, -125mmHg, and -250mmHg on the same subject with the left leg dressed in the NPWT dressing and the right leg acting as a control.

**Figure 5:**
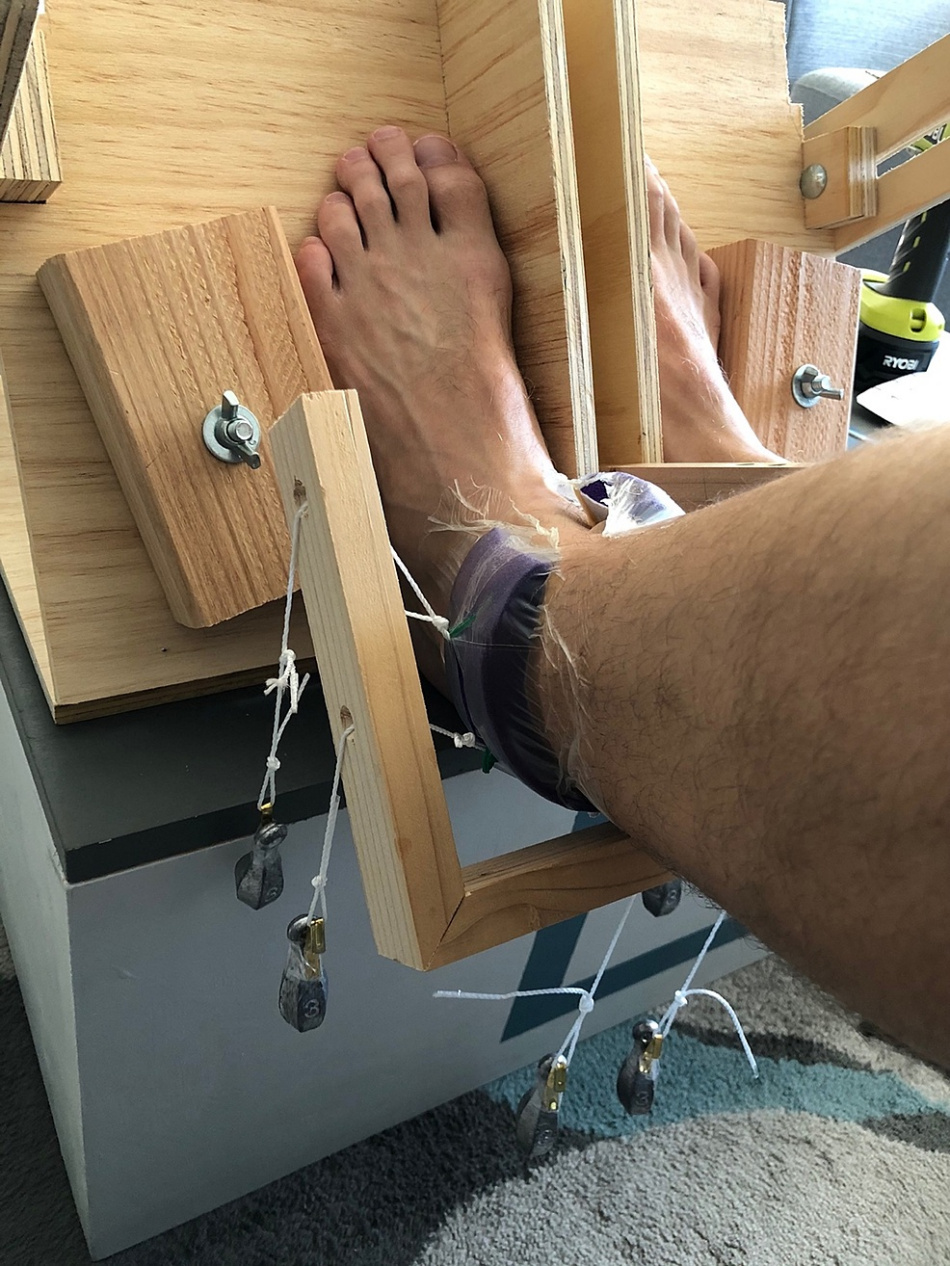
Five 85g lead weights were hooked onto loops of string that were tied onto the NPWT dressing. This provided a “lift-off” force to the dressing. Weights were used instead of negative pressure for the final experiment. NPWT: negative pressure wound therapy

**Figure 6:**
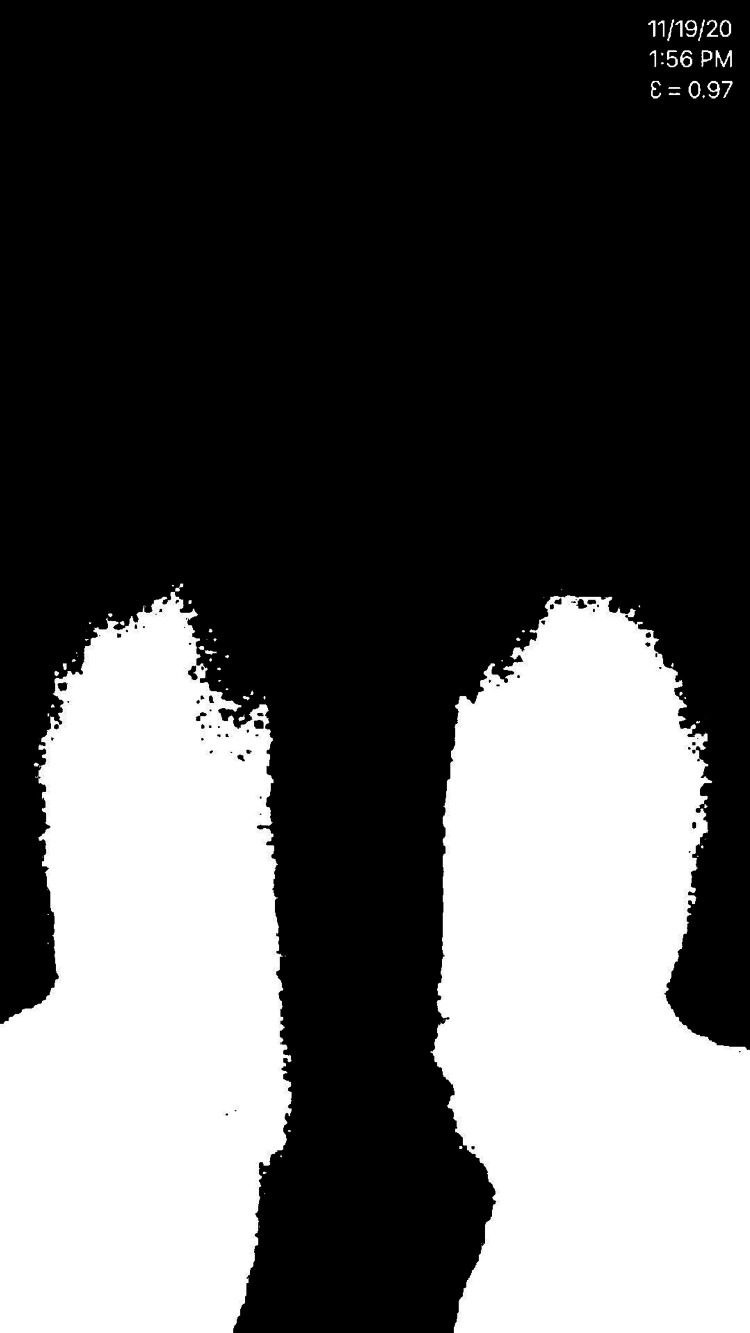
The thermal images were converted to a black and white photo utilizing the threshold function on ImageJ. The black and white pixels could then be counted to quantify the temperature of each foot.

To maximize any potential effect of the “lift-off” from the dressing, a third experiment was performed. The dressing used for this experiment was modified by securing string to five locations around the dressing. The string went through a frame which encircled the lower leg at the level of the dressing and were then tied in loops outside of the frame. Five 85g lead weights could then be hooked onto these loops to provide a “lift-off” force to the dressing. These weights were used instead of negative pressure for the final experiment (Figure 5). ImageJ was used to analyze the thermal images. First, the images were converted from a greyscale image to a black and white image by utilizing the threshold function on ImageJ (Figure 6). A threshold value was determined by selecting what appeared to be the warmest overall thermal image and increasing the threshold value until the amount of white in the process image plateaued. This ensured that the greatest potential change would be observed when comparing the coolest and the warmest thermal images. All the images underwent the same threshold changes by using a macro batch process. Next, the images were cropped in a batch process to include only the feet and reduce the potential of any extraneous thermal signals in the environment. The number of white pixels within each foot was determined by using a macro batch process to select a rectangular area around the foot to be analyzed and then counting the number of white pixels with the analyze particle function. This was repeated for both feet and the data was copied from ImageJ into Microsoft Excel (Microsoft Corporation, Redmond, USA) for analysis. The exposure of the thermal camera was kept constant throughout the experiments.

A paired t-test was used to determine whether the temperature of the left foot with NPWT on was significantly different from the temperature with NPWT off. If there was a significant difference (p<0.05), the p-value was calculated for the right foot during the same time period. If the difference was significant for the left and not for the right, the change in temperature could be attributed to the NPWT. If the difference during the time period was significant for both the left and right, the change was unlikely to be attributed to the NPWT. If the two time periods being compared did not have the same number of data points, the excess data points were omitted from the t-test calculation in order to run a paired t-test for all calculations.

## Results

**Figure 7:**
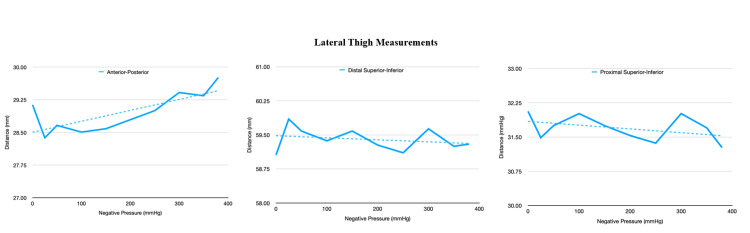
This figure demonstrates the change in the distance at the lateral aspect of the thigh at various negative pressures.

**Figure 8:**
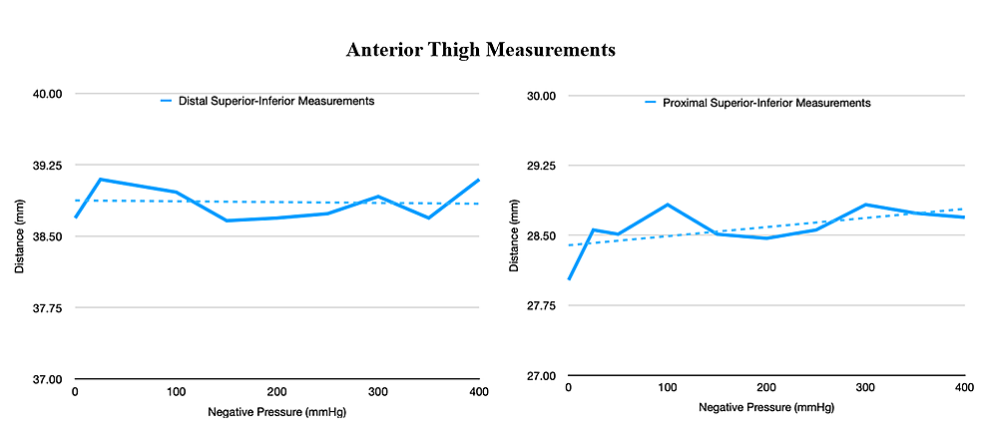
This figure demonstrates the change in the distance at the anterior aspect of the thigh at various negative pressures.

The first experiment demonstrated a notable change in the distance between the markers between the ends of the near-circumferential dressing (anteroposterior [AP] measurement). This distance increased as suction increased. This finding coincides with our previous study [18]. There was minimal change in the distance between the rest of the markers (Figures 7 and 8).

**Figure 9:**
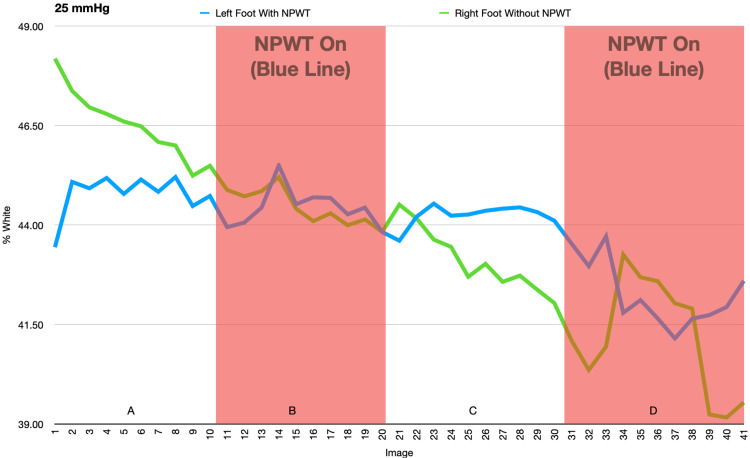
This figure demonstrates the change in percent white as a measurement of temperature as NPWT cycles on and off at -25mmHg. A and C: NPWT off, B and D: NPWT on. NPWT: negative pressure wound therapy

**Figure 10:**
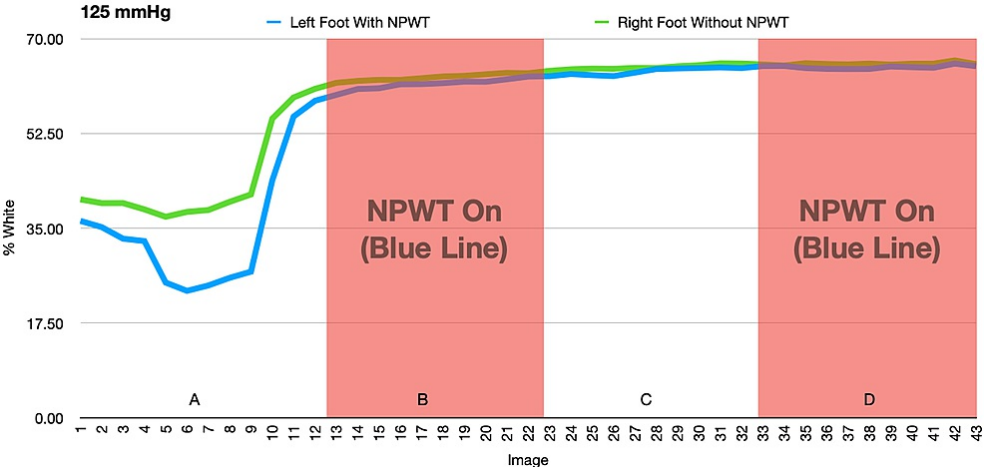
This figure demonstrates the change in percent white as a measurement of temperature as NPWT cycles on and off at -125mmHg. A and C: NPWT off, B and D: NPWT on NPWT: negative pressure wound therapy

**Figure 11:**
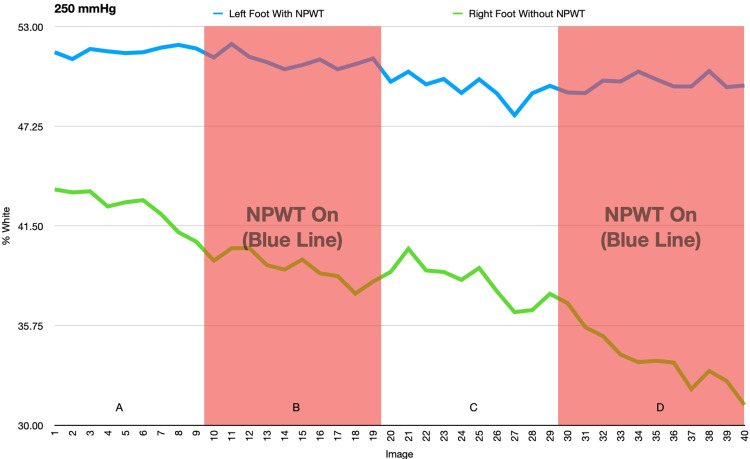
This figure demonstrates the change in percent white as a measurement of temperature as NPWT cycles on and off at -250mmHg. A and C: NPWT off, B and D: NPWT on. NPWT: negative pressure wound therapy

**Figure 12:**
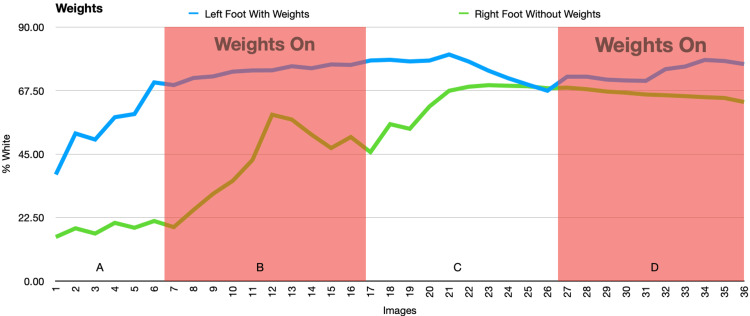
This figure demonstrates the change in percent white as a measurement of temperature as weights are on or off. A and C: weights off, B and D: weights on.

In the second and third experiments, there were significant changes in the temperature of the feet when the negative pressure was on or off, but these changes were mirrored by similar changes seen in the control foot. This suggests that the changes were due to acclimation of both feet or other external factors rather than an effect of the NPWT dressing (Figures 9-12 and Tables 1-4).

**Table 1:** Average percent white (-25mmHg) NPWT: negative pressure wound therapy

	Left Foot Average (with NPWT)	Left Foot Standard Deviation	Right Foot Average (no NPWT)	Right Foot Standard Deviation	Data Set	P-value
1-10 vac off (A)	40.710	0.525	42.292	0.877	Left Foot	
11-20 vac on (B)	40.398	0.477	40.402	0.450	A to B	0.068
21-30 vac off (C)	40.227	0.259	39.199	0.801	B to C	0.213
31-41 vac on (D)	38.735	0.834	37.733	1.452	C to D	<0.001
					Right Foot	
					A to B	<0.001
					B to C	<0.001
					C to D	0.005

**Table 2:** Average percent white (-125mmHg) NPWT: negative pressure wound therapy

	Left Foot Average (with NPWT)	Left Foot Standard Deviation	Right Foot Average (no NPWT)	Right Foot Standard Deviation	Data Set	P-value
1-12 vac off (A)	32.384	11.935	40.619	8.841	Left Foot	
13-22 vac on (B)	61.615	0.973	62.888	0.633	A to B	<0.001
23-32 vac off (C)	63.980	0.676	64.781	0.461	B to C	<0.001
33-43 vac on (D)	64.791	0.309	65.408	0.238	C to D	0.005
					Right Foot	
					A to B	<0.001
					B to C	<0.001
					C to D	<0.001

**Table 3:** Average percent white (-250mmHg) NPWT: negative pressure wound therapy

	Left Foot Average (with NPWT)	Left Foot Standard Deviation	Right Foot Average (no NPWT)	Right Foot Standard Deviation	Data set	P-value
1-9 vac off (A)	51.606	0.234	42.551	1.063	Left Foot	
10-19 vac on (B)	51.025	0.426	39.052	0.829	A to B	0.025
20-29 vac off (C)	49.444	0.695	38.211	1.149	B to C	<0.001
30-40 vac on (D)	49.741	0.422	33.801	1.668	C to D	0.367
					Right Foot	
					A to B	<0.001
					B to C	0.001
					C to D	<0.001

**Table 4:** Average percent white (with weights) NPWT: negative pressure wound therapy

	Left Foot Average (with NPWT)	Left Foot Standard Deviation	Right Foot Average (No NPWT)	Right Foot Standard Deviation	Data Set	P-value
1-6 vac off (A)	46.812	10.869	15.974	2.158	Left Foot	
7-17 vac on (B)	74.205	2.315	41.970	13.729	A to B	0.004
18-27 vac off (C)	75.381	4.337	62.910	8.405	B to C	0.559
28-37 vac on (D)	74.229	2.933	66.113	1.519	C to D	0.616
					Right Foot	
					A to B	0.022
					B to C	<0.001
					C to D	0.326

## Discussion

Current literature regarding the effect of NPWT on perfusion is mixed. One recent study by Ma et al showed that NPWT can preferentially enhance blood flow in a rat model [19]. Another study by Chen et al showed that NPWT may increase capillary caliber and blood volume by stimulating angiogenesis [11]. Some studies have suggested that a combination of microdeformation and macrodeformation can lead to an increase in pro-angiogenic growth factors promoting wound healing and recovery [20-22]. However, the macrodeformation due to the compressive forces of a circumferential NPWT has led to concern on whether this compression may negatively impact perfusion [23]. This was shown in a study by Shon et al which demonstrated decreased oxygenation saturation (SpO2) of the foot with an NPWT dressing [24]. On the other hand, a study by Aljomah et al in 2020 showed that a circumferential NPWT dressing around the upper arm did not decrease SpO2 in human subjects [25]. These opposing reports led to the conception of this study.

In the first experiment, the skin was noted to stretch in the open area between the short ends of the near-circumferential NPWT dressing. This coincides with our previous finding which demonstrated a “lift-off” force with a near-circumferential NPWT dressing. There were minimal changes to the skin around the other edges of the NPWT dressing suggesting that most of the “lift-off” force occurs as the dressing shrinks along its length rather than its width. 

In the second and third experiments, the temperature of both feet varied significantly but these changes in temperature were equally variant in conditions both with and without the NPWT dressing, suggesting that the NPWT had no effect on the temperature of the foot. Initially, a decrease in temperature was seen as a result of immobilization of the feet in an air-conditioned room. The subsequent increase in temperature was likely due to thermoregulation of the feet to the cold.

The results from the second and third experiments of this study can be interpreted in several ways. First, the lack of any significant changes in perfusion with a near-circumferential NPWT dressing may be due to a clinically insignificant change in the pressure of the extremity. Although decreased pressure was seen in the extremity analogue from our previous study, this effect may be dampened on a human extremity [18]. In particular, the distal lower leg may be suboptimal for showing this effect since this area has minimal compliant tissues such as fat and muscle and is mostly comprised of less compliant tissues like tendons. A more optimal area to test whether or not a near-circumferential NPWT dressing can change the pressure of an extremity would be around the thigh since it has the greatest surface area for contacting the NPWT dressing and has the most compliance. The reason why the distal lower leg was chosen for the second and third experiments, however, was because the temperature of the foot varies drastically, and we hypothesized that it would be the most sensitive to thermal imaging studies. Second, the lack of significant changes in the thermal images may not be due to a lack of changes in perfusion, but rather an inability of the thermal camera used in this study to detect such changes. Although the thermal camera used for this study was one of the best consumer-level thermal cameras, more advanced systems such as Stryker’s SPY-ELITE thermal imaging system (Stryker, Kalamazoo, USA) may have been able to detect changes that our camera could not. Third, the subject used for this study was a healthy adult male with no trauma or comorbidities. If a similar protocol had been used on a subject with a compromised lower extremity, results may have varied significantly either in favor of or against the use of NPWT dressings to increase perfusion.

The decision to use thermal imaging to measure perfusion was based on initial trial and error where we first used SpO2, transcutaneous partial pressure of oxygen (tcpO2), a temperature probe, and a laser vein finder to measure the perfusion of the foot under the same protocol as experiment two. However, we found no significant changes in SpO2, tcpO2, or temperature throughout the study. The laser vein finder proved to be an ineffective measurement for perfusion as the refresh rate of the laser projector led to shutter artifacts on the camera. Given that laser doppler validity has come into question and thermal imaging techniques have been previously used to assess perfusion, we thought thermal imaging would provide the most accurate and reliable measure of foot perfusion [12].

There are several limitations of this study. It was performed on only one healthy patient with no trauma or significant past medical history. Also, in experiment two, we saw an initial decline in the temperature of the foot at -125mmHg, which was thought to have been due to the prolonged immobilization period, causing the feet to get cold, followed by a compensatory increase in temperature as the body thermoregulated. The room temperature varied from 73°F for the -125mmHg trial to 69°F at the -25mmHg and -250mmHg trials. Thus, to limit this experimental variable, it would have been optimal to test these pressure differences under the exact same temperature conditions. Finally, we only tested a near-circumferential NPWT dressing since this was found to have the greatest effect on decreasing extremity pressure in our previous study [18]. To be more thorough, a fully-circumferential NPWT dressing could have been tested in this study as well. 

To our knowledge, this was the first study examining skin contraction or stretching around a near-circumferential NPWT dressing on a human subject. It is also the first study to utilize thermal imaging in conjunction with a near circumferential NPWT dressing. The methods for this study had several strengths as well. The 20-minute cycles of negative pressure used in the second experiment were based on several studies which have shown that acclimatization typically occurs within this time interval [7,13-14, 25]. All image processing was done in a batch format so the same settings were applied to all of the images. Motion was controlled for with a tripod and custom foot mount (Figure 3). Finally, to determine if the lift-off force generated from the NPWT dressing was not strong enough to lead to detectable changes in foot perfusion, weights were added to the dressing in the third experiment to maximize this potential lift-off force and improve the odds of noting any changes in foot temperature. 

Future studies are needed to further assess the effect of a near-circumferential NPWT dressing on extremity perfusion. To truly compare perfusion with and without a near-circumferential NPWT dressing, animal models could be utilized to create identical pathology and controls. Higher fidelity thermal cameras could be utilized but angiography would likely offer the most sensitive measurement of perfusion [26]. This methodology could also be repeated on multiple patients with various co-morbidities but it is difficult to have a valid control in these situations.

## Conclusions

Based on our findings, there appears to be a “lift-off” mechanism that may theoretically increase perfusion created by a near-circumferential NPWT dressing, but this study was unable to detect any changes in perfusion attributable to the NPWT dressing. This study has shown that for a healthy human subject, a near-circumferential NPWT dressing likely has no detrimental effect on perfusion when placed around the lower leg, however, we cannot definitively say how this dressing would affect a compromised extremity. Future studies are necessary to fully elucidate the effects of a near-circumferential NPWT dressing on extremity perfusion.
